# Bcr-Abl Allosteric Inhibitors: Where We Are and Where We Are Going to

**DOI:** 10.3390/molecules25184210

**Published:** 2020-09-14

**Authors:** Francesca Carofiglio, Daniela Trisciuzzi, Nicola Gambacorta, Francesco Leonetti, Angela Stefanachi, Orazio Nicolotti

**Affiliations:** 1 Dipartimento di Farmacia Scienze del Farmaco, Università degli Studi di Bari “Aldo Moro”, 70125 Bari, Italy; francescacarofiglio94@gmail.com (F.C.); daniela.trisciuzzi@uniba.it (D.T.); nicola.gambacorta1@uniba.it (N.G.); francesco.leonetti@uniba.it (F.L.); 2 Molecular Horizon srl, Via Montelino 32, 06084 Bettona, Italy

**Keywords:** Bcr-Abl, chronic myeloid leukemia, allosteric inhibitors, de novo design, artificial intelligence

## Abstract

The fusion oncoprotein Bcr-Abl is an aberrant tyrosine kinase responsible for chronic myeloid leukemia and acute lymphoblastic leukemia. The auto-inhibition regulatory module observed in the progenitor kinase c-Abl is lost in the aberrant Bcr-Abl, because of the lack of the *N*-myristoylated cap able to bind the myristoyl binding pocket also conserved in the Bcr-Abl kinase domain. A way to overcome the occurrence of resistance phenomena frequently observed for Bcr-Abl orthosteric drugs is the rational design of allosteric ligands approaching the so-called myristoyl binding pocket. The discovery of these allosteric inhibitors although very difficult and extremely challenging, represents a valuable option to minimize drug resistance, mostly due to the occurrence of mutations more frequently affecting orthosteric pockets, and to enhance target selectivity with lower off-target effects. In this perspective, we will elucidate at a molecular level the structural bases behind the Bcr-Abl allosteric control and will show how artificial intelligence can be effective to drive the automated de novo design towards off-patent regions of the chemical space.

## 1. Introduction

Bcr-Abl is a highly attractive target widely studied for the successful discovery of drugs for chronic myeloid leukemia (CML). However, the resistance phenomenon has raised several concerns and prompted the design of allosteric drugs [1]. Allosteric control implies the modulation of an enzyme by a small molecule binding at a site (allosteric site) other than the orthosteric site at which catalytic activity occurs [2,3,4]. The identification of allosteric sites is a real nightmare as these binding pockets are usually poorly accessible to current experimental methods being often hidden in less populated higher energy conformations [5,6,7,8]. The molecular perturbation induced by allosteric ligands determines not only the reorientation of the areas in the proximity of the allosteric site, but also promotes relevant conformational changes of the orthosteric site [9,10,11]. This intimate structural relationship between allosteric and orthosteric sites can be employed to circumvent issues typically inherent to the orthosteric pocket, such as the occurrence of point mutations, which are responsible for resistance phenomena. On the other hand, biasing allosteric sites can enhance target selectivity and lower off-target effects being their gene sequences fewer homologues compared to those of the orthosteric sites [12,13,14]. Keeping these in mind, the identification and characterization of allosteric sites represent a daunting task for the rational design of novel modulators, thus opening new fascinating perspectives in the discovery of new Bcr-Abl inhibitors based on allosteric control. In the present study, we will first discuss where we are, at a molecular level, by illustrating the successes and failures of the discovery of new Bcr-Abl allosteric inhibitors. Next, we will speculate about where we are going by reporting as new drug discovery technologies based on advanced molecular modeling and artificial intelligence can help medicinal chemists to properly address the rational de novo design in search of even new desirable chemotypes. In this respect, we herein propose a panel of potential Bcr-Abl allosteric inhibitors whose structures, generated by a creative drug discovery algorithm and biologically challenged by retrospective molecular docking screening, have never been encountered before to the best of our knowledge. Based on this wealth of information, we are confident that this study could be valuable to assist and drive the rational design of new allosteric inhibitors by exploring wider chemical space and fueling new research ideas.

## 2. Structural Bases for the Auto-Inhibition of c-Abl Tyrosine Kinase

Abl is a group of cytoplasmic tyrosine kinases consisting of two members, Abl (Abelson) and Arg (Abl-related gene), encoded by the ABL1 and ABL2 genes in humans, located on chromosome 9 and 1 respectively [15]. Nowadays, two different types of Abl tyrosine kinases are known. The first, termed as c-Abl, is the product of the mammalian proto-oncogene *c-abl* and is a ubiquitously expressed 140 kDa non-receptor tyrosine kinase [16,17] involved in the reorganization of the cytoskeleton after DNA damage and oxidative stress. The second, termed as v-Abl is the product of the viral gene *v-abl*, initially isolated from the Abelson murine leukemia virus [18]. Moreover, the Abl family is made up of two Abl isoforms (1a and 1b) and two Arg isoforms (1a and 1b). Both the 1b isoforms include an N-terminal myristoylated site, lacking in the 1a isoforms [19].

**Figure 1:**
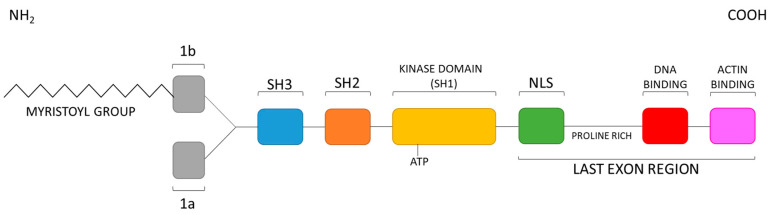
Abl domain order.

The crystallographic analysis of the cytoplasmic tyrosine kinase Abl shows that its regulatory site consists of the N-cap, the SH3 and SH2 domains, the kinase domain (KD), made by N- and C-lobes separated by the catalytic cleft, and the long C-terminal tails, termed as the last exon region [20]. Importantly, this domain order, as shown in Figure 1, is conserved among the Abl, Src (Sarcoma), Csk (C-terminal Src), Brk (Breast tumor kinase), and Tec (T helper cell differentiation) non-receptor tyrosine kinase families.

**Figure 2:**
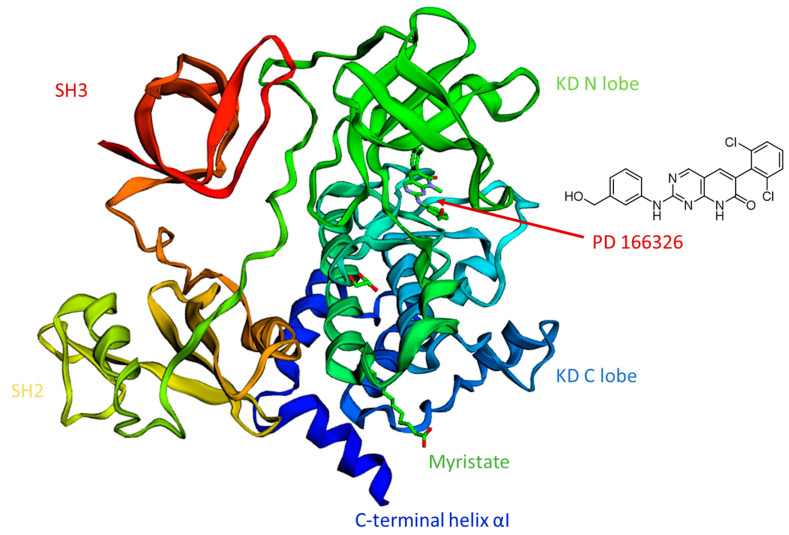
Auto-inhibited Abl in complex with the ATP competitive inhibitor PD166326 and myristate (PDB entry, 1OPK [20,27]).

The activity of Abl kinases is regulated by a sophisticated network of intramolecular interactions stemming from the Abl KD, which is responsible for the effective inhibition of tyrosine kinase activity. Notably, Abl is physiologically auto-inhibited by interactions of the SH3 and SH2 domains with the KD through its N- and C-lobes, respectively, as well as by the interplay of the myristoylated N-terminal (present in the Abl 1b splice variant) into a hydrophobic cavity of the KD C-lobe [20,21]. In this regard, data coming from X-ray of c-Abl complexed to PD166326 (an ATP, adenosine triphosphate, competitive inhibitor) and myristic acid (that is myristate at physiological pH) (PDB entry, 1OPK [22], shown in Figure 2), as well as from NMR studies [23] show the so-called assembled state of the Abl structure in the presence of myristate [24]. Similar assembled structures have so far been observed for the Src [25] and Btk (Bruton′s tyrosine kinase) [26] kinases.

In detail, the N-cap contains approximately 80 residues with a critical role in auto-inhibition. As mentioned before, the interaction between the SH3 and the SH2 domains facilitates auto-inhibition, involving the binding of the SH3 domain to the linker sequence (polyproline) between the SH2 and the KD. The interaction between the SH2 domain and the KD C-terminal lobe follows the first SH3-polyproline segment interaction, in order to form a clamp structure [21,22,28].

In the case of auto-inhibition, the myristoyl group (represented by myristate in the crystal) binds its so-called myristoyl pocket, which is an allosteric site made up by hydrophobic side chains situated in the KD C-lobes. Consequently, a bending of about 90° degree of the C-terminal helix αI of the KD was observed [20]. This event determines the docking of the SH2 domains to the base of the C-lobe of the KD, and stabilizes the clamp structure described before. Such conformational reorientation is responsible for the transition to an inactive auto-inhibited kinase state.

In the absence of the myristate, the C-terminal helix αI of the KD extends from residue Ser504 to Ser522, thus, leaving the subsequent residues disordered and determining an increased catalytic activity [29]. Indeed, when Abl is activated, SH2 and SH3 break away from the KD, thus it can bind various cellular targets [28] through intermolecular and intramolecular interactions. In particular, interactions between the Abl SH2 domain and the KD N-lobe have a critical role in leukemogenesis [30] and enhance in vitro kinase activity [31]. Even a partial, albeit persistent, disruption of the auto-inhibitory constraints, in which the SH3 and the SH2 domains play a significant role, results in oncogenic transformation. It was observed that Abl mutations that are close to the myristate pocket determined an increase of the kinase activity [32].

Summarizing, it is possible to recognize an Abl regulatory module (RM), constituted by the SH3, SH2, and N-cap domains, that is able to modulate KD activation by shifting from the inactive to active state [33]. When the RM docks at the back of the KD, thanks to the interactions of its SH2 and SH3 with C- and N-lobes, Abl is auto-inhibited by the penetration of the myristoyl group deep into its hydrophobic myristoyl pocket.

## 3. Bcr-Abl Tyrosine Kinase and Related Inhibitors

As known, the inadvertent activation of the Abl could cause several types of leukemia. In this respect, we will pay particular attention to the Chronic Myeloid Leukemia (CML). The etiology of this disease arises from a reciprocal chromosomal translocation involving the long arms of chromosomes 9 and 22 [34]. The t(9;22) translocation results in a genetic rearrangement, which fuses a segment of the breakpoint cluster region (*bcr)* gene from chromosome 9 to a region upstream of the second exon of the *c-abl* gene on chromosome 22, resulting in the development of the Philadelphia chromosome (Ph) that encodes the Bcr-Abl tyrosine kinase fusion protein. Physiologically, *c*-*abl* encodes a non-receptor tyrosine kinase that has tightly controlled activity in normal cells. Depending on the translocation breakpoint within the *bcr* gene, a protein of 210 kDa (termed Bcr-Abl p210) or 190 kDa (termed Bcr-Abl p190) can be expressed. The expression of p210 is the molecular hallmark of CML, whereas the expression of either p210 or p190 can be found in Ph+ Acute Lymphoblastic Leukemia (Ph+ ALL). In contrast to its proto-oncogenic counterpart c-Abl, Bcr-Abl displays constitutive tyrosine kinase activity [35], despite the fact that the KD amino acid sequence of the Abl segment of Bcr-Abl is identical to that of c-Abl. Further studies will be necessary to better understand the reason for this intense catalytic activity, which plays an essential role in carcinogenesis.

**Figure 3:**
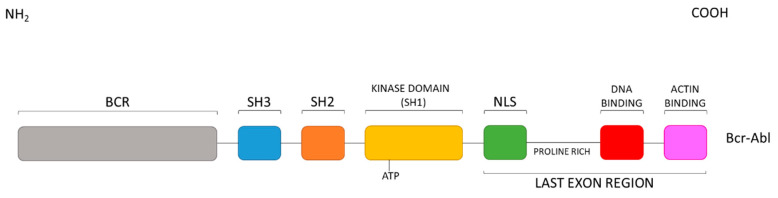
Bcr-Abl domain order.

Focusing the attention on the fusion protein Bcr-Abl, in this specific case, oncogenic activation is driven by the fusion of Bcr with c-Abl at its N-terminus (Figure 3) [21,36]. Indeed, Bcr-Abl is not myristoylated because of the lack of the first Abl exon, and the disruption of its regulatory mechanism by pertinent auto-phosphorylation results in uncontrolled oncogenic activity responsible for CML [37].

For the reasons above discussed, the inhibition of the tyrosine kinase Bcr-Abl activity by tyrosine kinase inhibitors (TKIs) is an important goal for CML therapy and depends on the state of activation in which the kinase exists at the time of the binding of the inhibitors [38]. Recently, TKIs were classified in four types according to their binding mode, as follows: (a) Type I TKIs interact with ATP binding site in the so-called DFG-IN (see below) active conformation; (b) type II TKIs occupy the binding pocket in the so-called DFG-OUT inactive (see below) conformation; (c) type III TKIs bind to an allosteric pocket near the ATP pocket; (d) type IV TKIs can bias to a pocket away from the ATP one, but always in the KD.

Recently, allosteric inhibitors binding outside the KD, such as those targeting the pseudokinase (kinase that does not conserve the catalytic motifs and for this reason preannounced as catalytically inactive) domain, have been discovered [39]. Lu et al. designated two new types of inhibitors, named as types VI and VII, targeting the pseudokinase domain or an extracellular domain as binding sites, unlike from the originally conserved allosteric inhibitors (types III and IV) approaching the KD [40].

## 4. Bcr-Abl Orthosteric Inhibitors

Type I, II, III, and IV TKIs bind to the KD of Bcr-Abl, conserved from Abl kinase. For this reason, these inhibitors may be studied by employing the currently available Abl crystal structures. In detail, to better understand differences among type I and II TKIs, it must be considered that the Abl KD is bilobal, comprising an N-terminal lobe (N-lobe), and a larger C-terminal lobe (C-lobe). Furthermore, the peptide substrate binds primarily to the C-lobe, while the ATP binds the cleft between the two lobes through two hydrogen bonds involving the adenine of ATP and the main chain atoms of the so-called interlobe connector (i.e., the hinge) linking the N- and C-lobes. Importantly, a so-called gatekeeper residue, Thr315, is situated at the back of the ATP binding pocket. It confers specificity to the kinase and represents a determining factor for the binding of inhibitors [41]. This residue may also undergo a point mutation, becoming responsible for the resistance to TKIs of leukemia patients.

**Figure 4:**
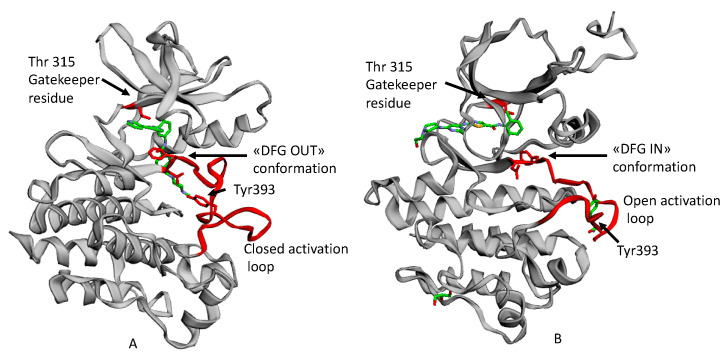
An example of ATP competitive inhibitors: (**A**) X-ray solved the structure of Abl KD in complex with imatinib (PDB entry, 1IEP [29]) with “DFG-OUT” conformation and closed activation loop; (**B**) X-ray solved the structure of Abl KD with dasatinib (PDB entry, 2GQG [43]) with “DFG-IN” conformation and open activation loop [27,44] (The picture is taken from Carofiglio et al. [45]).

The most flexible segment in KD is the so-called activation loop that stems from the C-lobe and plays a central role in activation. The loop is centrally located and contains a preserved DFG motif (Asp381-Phe382-Gly383 in Abl) at N-terminus, while the middle portion contains a tyrosine (that is Tyr393), or a serine/threonine, phosphorylated for activation. When the kinase is in its active state, the activation loop is in an open or extended conformation wherein the aspartate of the DFG motif points “IN” towards the ATP binding site and coordinates to the catalytic Mg^2+^ ion(s) and the C-terminal portion of the loop forms part of the platform for peptide substrate binding. Specifically, when the motif is in this DFG-IN conformation, the Asp residue is correctly oriented to provide a catalytically competent active conformation. Instead, in the DFG-OUT conformation, the Asp residue is directed away from the active site, its position is swapped in a crankshaft-like motion also known as DFG-flip with the Phe residue, which in the latter conformation opens a hydrophobic pocket between the active site and the αC helix. This pocket opens when the DFG flips. From long unbiased molecular dynamics (MD) simulations [42] carried out during several computational studies by Shan et al. a notable result was the suggestion that the protonation state of Asp381 in the DFG motif could serve to promote the “IN” to ”OUT” conformational transition (see Figure 4).

Imatinib was the first ATP competitive inhibitor, which specifically blocks the binding of ATP to the catalytic site, leading to the inactivation of Bcr-Abl, and eventually, to the amelioration of CML pathological conditions by improving the overall survival of patients [46]. In particular, imatinib binds the Abl KD in its inactive state that is the “DFG-OUT” conformation.

However, the occurrence of point mutations has caused acquired resistance and reduced sensitivity to imatinib. The most common mutation in BCR-ABL occurs in the KD, where more than 90 mutations have been described. The most frequent mutations are: G250H, Q252H, Y253H, E255K, T315I, and F359V [36]. The T315I, involving the gatekeeper function, represents the most widespread and hard to tackle mutation of CML. Unfortunately, the occurrence of T315I point mutation, makes Bcr-Abl resistant also to some of the second-generation TKIs, which are instead effective in the presence of other mutations.

**Figure 5:**
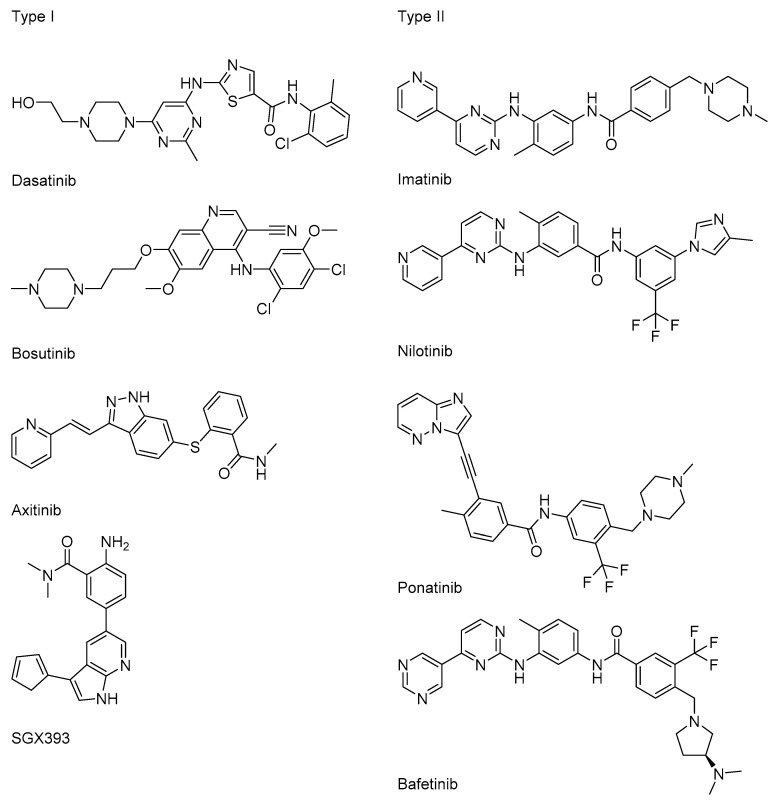
Bcr-Abl ATP competitive inhibitors.

Undoubtedly, the successful introduction of imatinib as a Bcr-Abl inhibitor has revolutionized the CML treatment. Still, the occurrence of the mutations above mentioned has sparkled the interest to search for inhibitors capable of overriding it. For this reason, nilotinib, dasatinib, bosutinib, and bafetinib have been developed as more potent Bcr-Abl inhibitors, and some of them, like nilotinib and dasatinib, gained regulatory approval for second-line use in CML patients resistant to imatinib [47,48,49,50,51]. Among the drugs that show activity towards T315I, there are ponatinib (AP2453, Ariad Pharmaceuticals, Cambridge, MA, USA), axitinib, and SGX393 (Eli Lilly, Indianapolis, IN, USA) [52,53]. Likewise imatinib, they are ATP competitors, but unconstrained by the steric clash of the I315 side chain. The most known Bcr-Abl ATP competitive inhibitors (type I and type II) are shown in Figure 5.

Fundamental to understand how ATP competitive inhibitors were able to influence the assembly state of Abl via the activation loop conformation was a paper recently published by Sonti et al., in which authors underlined that type I TKIs induce an assembled state of the protein, while type II TKIs induce a disassembled state by pushing the kinase N-lobe toward the SH3 domain trough the activation and ATP binding loops [24].

## 5. Bcr-Abl Allosteric Inhibitors

The rational design of allosteric inhibitors, although difficult, can provide many advantages, such as avoiding the occurrence of resistance phenomena, due to orthosteric pocket point mutations, greater selectivity and less off-target effects. This is because gene sequences of the allosteric sites are fewer homologues than those of the orthosteric site [12,13,14]. As a result, allostery could be considered a very useful tool to rationally improve drug discovery [5,54,55,56,57,58,59]. In this scenario, the pocket for myristate binding on Bcr-Abl could be considered as an additional target site for the rational design of site-specific molecules capable of mimicking the myristate binding, and thus, to promote auto-inhibitory regulation [21]. Of course, these inhibitors are expected to be ATP non-competitive since they are supposed to mimic both the position and function of myristoyl group [60], thereby decreasing Bcr-Abl aberrant kinase activity [40].

**Figure 6:**
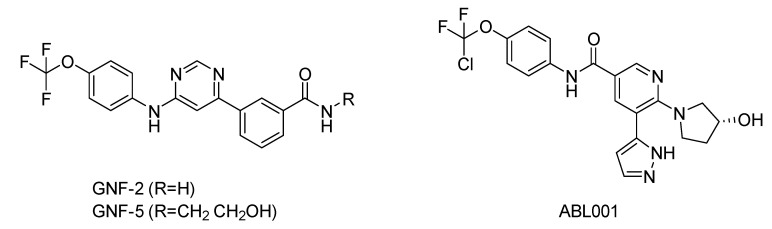
Bcr-Abl allosteric inhibitors.

**Figure 7:**
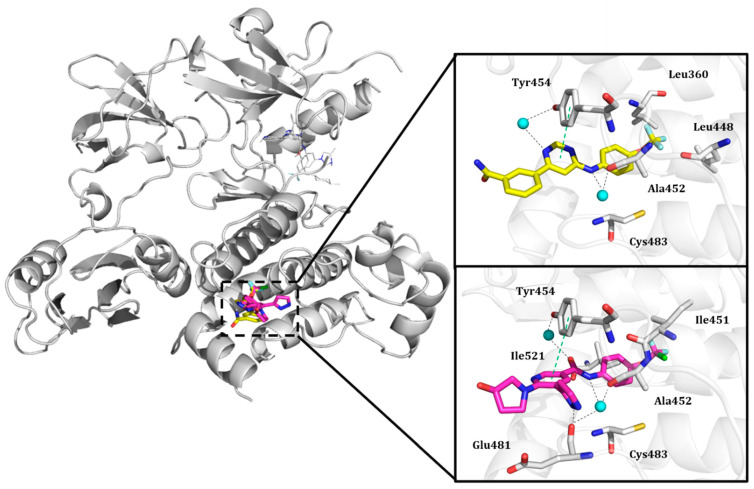
Details of the molecular interactions between GNF-2 and ABL001 and the myristate binding pocket reported in the top and bottom zoom-in views, respectively. The key residues are reported in sticky representation. The water bridge molecules are shown as cyan spheres. Black and green dashed lines indicate hydrogen bonds and π-π interactions, respectively.

Shown in Figure 6, GNF-2 and GNF-5 were the first to be identified as Bcr-Abl type IV TKIs binding the myristoyl pocket site at the C-terminus of Abl KD [60,61,62]. GNF-2, in combination with imatinib, showed a synergistic inhibitory effect of Abl [60,61,62,63]. GNF-2 binds to the myristoyl site with its trifluoromethoxy group buried deeply in the hydrophobic pocket. As shown in Figure 7, GNF-2 is able to establish hydrophobic bonds with Leu448, Ala452, and Leu360. The pyrimidine ring nitrogen atom forms an HB with Tyr454 mediated by one bridge water molecule and its amine group with Ala452. This compound specifically inhibits the proliferation of Ba/F3 (a murine interleukin-3 dependent pro-B cell line) cells transformed to express Abl kinase, while displaying no activity against non-transformed cells [61]. Unfortunately, GNF-2 and GNF-5 lost their potency against Ba/F3 cells presenting Bcr-Abl mutants, and in particular, those including the gatekeeper T315I mutation. A combination of GNF-5 and nilotinib, an ATP competitive Abl inhibitor, led to additional inhibitory activity in biochemical and cellular assays against the Bcr-Abl T315I mutant, displaying good efficacy in a murine bone marrow transplantation model. The results suggest that allosteric myristate binding site inhibitors of Bcr-Abl combined with ATP competitive Bcr-Abl inhibitors may effectively overcome clinically acquired resistance in the treatment of CML resulting from the Bcr-Abl T315I mutation [64].

Very recently, Novartis reported the discovery of ABL001 (also known as asciminib), which is the first type IV allosteric Bcr-Abl TKI to enter in clinical trials. Its discovery was performed by employing a fragment-based approach and supported by an NMR conformational assay [65].

ABL001 binds to the allosteric myristoyl pocket site of Abl kinase, likewise GNF-2. It mimics the myristate substrate and potently binds to the myristoyl pocket of Abl with K_d_ values from 0.5 to 0.8 nM. Importantly, ABL001 is active in the low nanomolar range against all catalytic ATP site mutations in Bcr-Abl, including the gatekeeper T315I mutation [66].

Unfortunately, many reports demonstrated that some mutations in the myristoyl binding site (P465S, V468F, I502L, C464W, and A337V) determined resistance to ABL001. Cell proliferation assays were performed on these mutants, and decreased activity of ABL001 was observed, while the ones of classical orthosteric drugs kept unchanged [67,68].

The combination use of ABL001 and ATP competitive Bcr-Abl inhibitors (such as nilotinib and ponatinib) was effective in overcoming resistance problems, due to mutations both at the ATP binding site or the ones observed near the allosteric myristoyl binding pocket [68,69]. ABL001 is currently in clinical trials for the treatment of CML and Ph+ ALL as a single agent and in combination with imatinib (NCT03106779 and NCT03578367, respectively).

## 6. Non-Small Molecule Bcr-Abl Allosteric Inhibitors

It is worth to emphasize that Bcr-Abl has multiple protein domains. In particular, through extensive mechanistic and structural studies, an interaction between the SH2 and the KD seems to be required for full kinase activity. In this respect, it was found that the point mutation I164E inhibits the enzymatic activity by disrupting the interface between these two domains, and can thus trigger oncogenesis [70,71]. Based on this evidence, the interface between the SH2 and KD could be a potential target for the allosteric kinase inhibition. To this end, a monobody termed as 7c12, able to approach the kinase binding surface of the Abl SH2 domain has been employed. Given its moderate affinity, and hence, moderate biological effects in vitro and in the cell, 7c12 was fused with another monobody, known as HA4, able to bind to a different region of the SH2 domain, namely, the binding site for phospho-Tyr-containing ligands [72]. This tandem fusion monobody 7c12-HA4 proved to successfully interfere with the occurrence of intramolecular interactions between the SH2 and KD. This mechanism determined the suppression of the Bcr-Abl dependent oncogenic transformation of mouse bone marrow cells through the Bcr-Abl kinase activity inhibition and induced apoptosis in human cells isolated from CML patients [71]. In particular, HA4 inhibits the interaction of Abl SH2 with its phosphor-Tyr-containing ligands and the consequent phosphorylation of Bcr-Abl substrates [72]. For all these reasons, the results of the trial supported that the SH2-kinase interface could be a druggable site in CML patients.

## 7. In Silico Approaches for the Design of Allosteric Inhibitors

In silico methods can play a crucial role in understanding the function of allosteric regulatory sites, and thus, to address the rational design of new potential allosteric inhibitors. In this respect, the most popular approaches rely on structure- and ligand-based strategies. The former is employed when the crystallographic solved structures or homology models of a given target are available. The latter is applied if structural data information is missing, and thus, structural similarity towards active known molecules becomes pivotal to carry studies, such as quantitative structure-activity relationship (QSAR) and pharmacophore modeling [73,74,75,76,77,78,79].

These computer-aided strategies are widely implemented in several free available web-server tools. An example is given by AllositePro version 2.10 webserver (http://mdl.shsmu.edu.cn/AST/), based on a method for predicting allosteric sites using a support vector machine based on topological and physiochemical pocket features combining with perturbation analysis [80]. Alternatively, CavityPlus (http://www.pkumdl.cn:8000/cavityplus/index.php) identifies putative binding sites located at the surface of a given protein structure and ranked according to druggability and ligandability parameters [81]. Another successful implementation for allosteric drug discovery is the Kinase Atlas server web tool (https://kinase-atlas.bu.edu/), a curated database of mostly unexplored allosteric sites [82]. The collection is built on crystallographic data of 4910 PDB structures of 376 distinct kinases. The so-called binding hot spots are identified by FTMap, an algorithm making use of small organic molecules as probes [83].

However, an intrinsic limitation concerned with structure-based strategies is that of the targeting flexibility. This becomes particularly important given that the allosteric inhibitors can induce conformational changes involving relevant structural reorganization (e.g., the orientation of the activation loop for the Abl kinase). To face this issue, different integrated in silico approaches can be used. In the paper of Singh and Coumar [84], an ensemble docking-based virtual screening was performed on the Abl myristoyl binding site by using a database of about 14,400 compounds. This strategy leads to select a set of seven compounds as putative candidate allosteric site modulators provided with higher docking scores than the co-crystallized allosteric inhibitor GNF-2, at least in three out of the four virtual screenings.

Although these computational techniques are low computationally demanding, they are however, inadequate to properly understand the intimate nature of allostery and its mechanism of action. In this respect, MD represents a valuable tool to study the evolution of biological systems in a range of time appropriate for the occurrence of particular events. In the case of Abl, the MD plays a predominant role in providing valuable information concerning kinase conformational states elucidating the molecular interactions underpinning molecular recognition and free-energy profiles inaccessible to current experimental methods.

**Figure 8:**
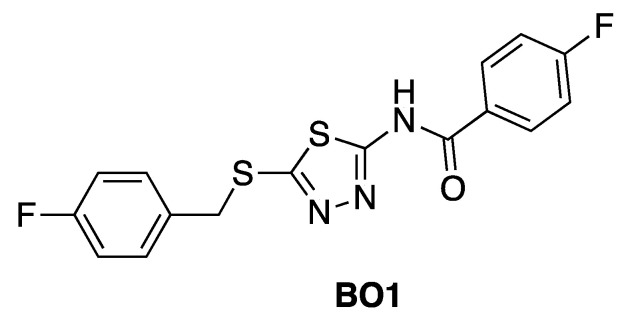
Structure of the ATP competitive/mixed inhibitor of Abl BO1.

For instance, in the study of Fallacara et al. [85], MD simulations in combination with molecular mechanics generalized Born surface area (MM-GB/SA) analyses were performed on the Abl wild type and gatekeeper mutant T315I in complex with two myristate binding pocket inhibitors, GNF-2 and BO1 (ATP competitive/mixed inhibitor of Abl wild type and purely noncompetitive ATP inhibitor in the case of Abl T315I) (Figure 8) [86,87,88].

Comparing with the myristate, GNF-2 induces the stabilization of the Abl wild type induced by an intramolecular interaction between kinase and SH2-SH3 domains, which is completely absent in the mutant protein. Moreover, the authors demonstrated that BO1 is able to establish a stable interaction within the myristoyl site of both Abl wild type and T315I through the formation of the compact conformation of the enzyme. Moreover, MD simulation and MM-GB/SA calculations can be used to understand the drug resistance mechanism for a given promising allosteric inhibitor. In a recent study, these in silico techniques unveiled an adversely influence on the binding of the well-known drug ABL001 to Abl myristic allosteric binding pocket, due to a lower contribution of the nonpolar interactions occurring in the two mutants (i.e., I502L and V468F) inducing a drug resistance during the clinical trials [89]. Alternatively, MD simulations can be combined with classical structure-based approaches to identify novel allosteric modulators. For instance, Banavath et al. [90] suggested seven lead compounds as promising drug candidates against both wild type and T315I mutant Abl by using a virtual screening approach based on molecular docking analysis integrated with MD simulations.

Nevertheless, the advent of artificial intelligence has enhanced drug discovery potential. In this scenario, approaches based on machine learning represent promising solutions for driving lead generation and lead optimization bypassing limitations posed by experimental methods. Such innovative methods are now widely employed in several scientific areas by academic researchers or pharmaceutical companies for their capability to generate high predictive models, which learn from the huge amount of data currently available [91]. In this regard, the machine learning methods have been fairly adapted to find new Bcr-Abl allosteric inhibitors. For instance, in the work of Bajorat et al., accurate and stable models have been developed based on random forest, support vector machine, and deep neural network algorithms [92]. The models are trained on a large database of compounds playing with different binding modes. The obtained global and balanced models can distinguish allosteric from non-allosteric kinase inhibitors with similar yet distinct mechanisms of action. These models results are very attractive because they can be used to explore new original scaffolds, and thus, move into a new possible off-patent chemical space.

In this perspective, we will illustrate two in silico strategies aiming to investigate the Abl allosteric binding pocket on one side and to design new potential small-molecule allosteric TKIs on the other. Specifically, the first analysis involved a structure-based strategy focused on the myristoyl binding site in order to elucidate the most representative allosteric residues and their energetic contribution. The second case study exploited an *in-house* automated generative machine learning algorithm able to design a library of new potential selective TKIs with desired properties, which can be easily set by the user [93].

### 7.1. Case Study I: Molecular Interaction Fields Analysis

As known, the structural diversity of the allosteric sites makes allosteric TKIs potentially higher selective with respect to orthosteric TKIs. In this scenario, targeting allosteric sites can be considered a novel trick requiring more in-depth analysis for their identification and characterization. The ultimate goal is to exploit the information concerning with allosteric binding site (i.e., the energetic contribution of the so-called hot spots residues) to optimize known drugs or to discover new potential compounds provided with new chemotypes.

In this present study, we carried out an in silico investigation of the Abl myristate binding site by analyzing the molecular interaction fields (MIFs) generated on the X-ray crystal structures of Abl by using the FLAP (Fingerprints for Ligands and Proteins) algorithm, which is developed and licensed by Molecular Discovery Ltd. (www.moldiscovery.com) [94,95]. FLAP explores the protein cavities whose 3D structures are known on the basis of the shape similarity and irrespective of the primary structures.

In particular, FLAP includes the automatic preparation of protein structure data, identification of binding sites, and comparison of the pockets by aligning the residue sequences or directly matching the MIFs. Additionally, FLAP employs MIFs generated through the GRID force field to evaluate the type, strength, and direction of the interactions that a molecule can establish [96].

In order to identify a broader spectrum of the protein residues and their energetic contribution in the myristoyl pocket, three X-ray crystal structures of Abl co-crystallized with myristic acid ligand (PDB entry, 1OPK [20], resolution 1.80 Å) and two allosteric inhibitors, GNF-2 (PDB entry, 3K5V [60], resolution 1.74 Å) and ABL001 (PDB entry, 5MO4 [67], resolution 2.17 Å), have been retrieved from the Protein Data Bank (PDB).

The protein residues were first processed using the *Fixpdb* tool, and all water molecules and cofactors were filtered. Specifically, only two water bridging molecules having a functional role have been retained for the complexes co-crystallized with the two inhibitors, as suggested in Reference [40]. The procedure implies the embedding of the target protein into a 3D grid centered on each co-crystallized ligand. The algorithm, thus, identifies the pocket points of the Abl myristic binding site using three GRID probes, which are CRY, N1, and O, to compute hydrophobic, HB acceptor, and HB donor interactions, respectively. In particular, all pocket points were detected by the probes focusing on grid points located within a distance of 2 Å from the closest ligand atom.

**Figure 9:**
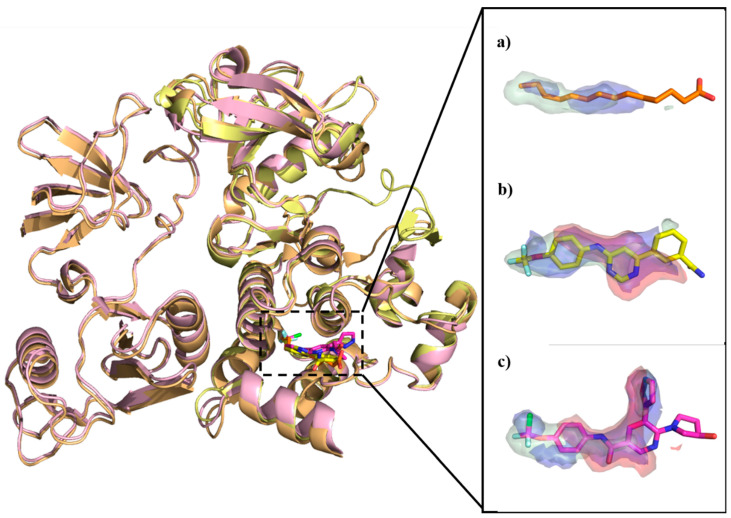
Superposition of Abl crystal structures co-crystallized with (**a**) the myristic acid (PDB entry, 1OPK [20]) and two allosteric inhibitors, (**b**) GNF-2 (PDB entry, 3K5V [60]), and (**c**) ABL001 (PDB entry, 5MO4 [67]) rendered in orange, yellow and pink cartoon respectively. In the insert, GRID MIFs probes for each crystal structure corresponding to hydrophobic, HB acceptor, and HB donor interactions are displayed in green, blues, and red surface, respectively. A cut-off value of –1.5 kcal/mol, −4.5 kcal/mol, and −3.0 kcal/mol is set for CRY-, N1-, and O-GRID probes, respectively.

As shown in Figure 9, the superposition of the three Abl crystal structures co-crystallized with the natural ligand myristic acid ligand (PDB entry, 1OPK [20]) and two allosteric inhibitors (Figure 9a), GNF-2 (PDB entry, 3K5V [60]) (Figure 9b) and ABL001 (PDB entry, 5MO4 [67]) (Figure 9c) is depicted. In the insert, GRID MIFs probes for each crystal structure expressing hydrophobic, HB acceptor, and the HB donor interactions associated with their co-crystallized cognate ligands are also shown.

A list of key residues of Abl allosteric pocket is obtained by means of GRID MIFs quadruplets for each cognate ligand matching, according to the respective GRID probe considered as summarized in Table S1 of Supporting Information. At first glimpse, protein residues of the complex co-crystallized with the myristate ligand are visited by both the complexes co-crystallized with two inhibitors adding the contribution of the HB donor interactions (i.e., O-GRID probes).

The obtained energetic values are confirmed by X-ray crystallography data showing a relevant role by Leu448, Ala452 and Leu360 that constitute the hydrophobic pocket in all the three crystal structures. Notably, the role of the two water molecules confirms to be relevant for the interaction with the two allosteric modulators with high energetic values for the Abl cavities co-crystallized with inhibitors. In the case of GNF-2, the nitrogen atom of the pyrimidine ring of the inhibitor forms an HB contact with Tyr454 mediated by water molecule. At the same time, the amine group also forms an HB network with Ala452 through another water molecule. For the allosteric modulator ABL001, the water molecules mediated two HB networks with Tyr454 on one side and Glu481 on the other side. The GRID analysis can also provide additional information so far, to our knowledge, never investigated. For instance, Arg351 plays as a strong, energetic HB donor contributor (see O-GRID-probe) in the allosteric pocket with a value equal to −4.683 kcal/mol in case of the Abl crystal structure co-crystallized with the ABL001. Similarly, a significant energetic contribution is associated with Ala356 as HB acceptor (N1-GRID probe) in all the three crystal structures. This finding may suggest the existence of a potential cavity to sample by optimizing known allosteric inhibitors or by de novo design.

### 7.2. Case Study II: de novo Drug Design Based on Artifcial Intelligence

Herein, we illustrate as the use of artificial intelligence in drug discovery can rationally address the de novo design of a targeted chemical library of inhibitors tailored to engage the myristoyl binding pocket. The quality and goodness of the de novo designed inhibitors towards the putative Abl allosteric pocket have been retrospectively assessed by employing molecular docking. In this study, recurrent neural networks were used to generate new molecules whose potential towards the myristoyl binding pocket was optimized by considering two easy interpretable molecular descriptors, that are the molecular weight (MW) and the logP, and the level of similarity with respect to ABL001, taken as a reference compound. The de novo designed compounds are sampled in a range of values reported in Table S2 of Supporting Information. The learning curves indicating the progress of the average of the S(x) fitness values of the pair based multi-objective algorithm is shown in Figure S1 of Supporting Information.

According to the ability to pass structural alert filters (see Table S3 of Supporting Information) equal to 82.8%, the whole Bcr-Abl library was thus further processed to discard those compounds potentially unsuitable for in vitro testing. Finally, molecular docking simulations have been retrospectively employed on a pool of 826 compounds (hereafter referred to as Bcr-Abl.lib) in order to inspect the molecular interactions of the de novo generated potential Bcr-Abl inhibitors. In this respect, the X-ray solved crystal structures of Abl co-crystallized with allosteric inhibitors ABL001 was retrieved from the Protein Data Bank (PDB entry, 5MO4 [67]). The X-ray protein structure was processed using *Protein Preparation Wizard* [97] available in the Schrodinger suite in order to remove co-crystalized water molecules, add hydrogen atoms, correct the protonation states or incomplete amino acid side chains and carry out energy minimization. Specifically, two bridging water molecules have been retained for their functional role to form two HBs with the ABL001, as suggested in Reference [40]. All the compounds of de novo targeted chemical library were thus prepared for docking simulations by employing the *LigPrep* tool [98] to properly generate all the possible tautomers and ionization states at a pH value of 7.0 ± 2.0. Finally, molecular docking was performed applying standard precision default settings available in GLIDE by automatically centering a cubic grid box with an edge equal to 16 Å on the co-crystallized cognate ligand ABL001 [99,100]. The reliability of docking simulation protocols was preliminary challenged by computing the root mean square deviation (RMSD) values (see Figure S2 of Supporting Information) that is as good as 1.020 Å. The docking results of de novo generated molecules were analyzed in comparison with the posing and scoring of the co-crystallized cognate ligands ABL001 in the allosteric binding pocket. The 15 top-ranked representative examples taken from Bcr-Abl.lib are shown in Table 1. The potential inhibitors include an aromatic amide scaffold, likewise the ABL001 inhibitor, to ensure the HB interactions with the Tyr454 e Glu381 mediated by water molecules. Notably, the identified compounds are novel, unique, and patentable compounds. The entire SMILES list of compounds, along with the docking score values, is reported as Supporting Information (see File_SI.csv). Three terms were mostly considered to evaluate the goodness of our analysis: The S(x) fitness values, the docking scores, and the chance of interacting with key binding site residues.

**Figure 10:**
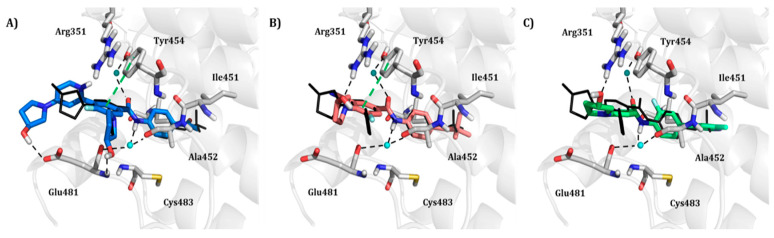
Molecular interactions between Bcl-Abl (PDB entry, 5MO4 [67]) and the docking top-scored de novo generated compounds taken from Bcr-Abl.lib referring to the compound Bcr-Abl.lib_01 (**A**), Bcr-Abl.lib_02 (**B**) and Bcr-Abl.lib_03 (**C**), respectively. The water bridge molecules are shown as cyan spheres. ABL001 is depicted in the black wireframe. Black and green dashed lines indicate hydrogen bonds and π-π interactions, respectively. For the sake of clarity, only polar hydrogen atoms are shown.

Hence, molecular docking simulations have been employed to inspect the molecular interactions of the de novo generated potential Bcr-Abl allosteric inhibitors. Interestingly, three molecules returned a docking score higher than the co-crystallized allosteric inhibitor ABL001, which is equal to −10.298 kcal/mol. As shown in Figure 10, the three top-ranked compounds taken from Bcr-Abl.lib are depicted in order to evaluate the interactions with allosteric cavities residues.

Worthy of mention, the three docking top-scored compounds showed a posing and a scoring comparable to ABL001 by experiencing very similar molecular interactions at the myristate binding site, thus confirming the high quality of the obtained results. As shown in Figure 10, all the amidic groups of the compounds can make HBs with Tyr454 mediated by water bridge molecule and two HBs with the backbone of the Glu481 and Ala452 mediated by another water molecule.

Bcr-Abl.lib_01 returns a docking score value equal to −10.704 kcal/mol. Notably, an HB can also occur with the carboxylic group of Glu481 through the hydroxyl group of pyrrolidine moiety (Figure 10A), thus explaining the higher docking score values of this candidate TKI with respect to the well-known inhibitor ABL001. It can also experience a π–π stacking with the side chain of Tyr454, as observed in the case of ABL001.

Bcr-Abl.lib_02 and Bcr-Abl.lib_03 returned a docking score equal to −10.338 kcal/mol and −10.305 kcal/mol, respectively being the hydroxyl group of pyrrolidine moiety involved in HBs with the side chains of Arg351 (Figure 10B,C). Although this interaction is missing in the case of ABL001, Arg351 can be an interesting allosteric cavity residue, as also previously suggested by FLAP pocket analysis.

## 8. Conclusions

Where are we and where are we going? Despite our best intentions, these questions are still there. Having said that, we are confident that artificial intelligence is indeed a very powerful tool to address drug discovery by unveiling the causative, although latent relationships existing between chemical and biological sides [101,102]. Importantly, the de novo drug design guided by artificial intelligence is not a mere decorative option, but rather an unprecedented chance to explore the chemical space in search of new chemotypes ideally optimal for specific drug targets. As shown for the de novo design of new Bcr-Abl allosteric inhibitors, the user can easily set appropriate similarity cut-offs and several physicochemical parameters to generate a targeted chemical library of compounds whose effectiveness in terms of chemical feasibility, validity, compliance to toxicity alerts, and patentability can be easily tuned. In this respect, retrospective molecular docking simulations can further support the in silico trials to rationally prioritize compounds for experimental testing [103]. Indeed, the informed use of artificial intelligence can enhance the knowledge-based human intuition by directing research towards unexpected successful results [104]. Last, but not least, artificial intelligence is certainly effective in inspiring the experimental work with low-cost ideas, as shown for the automated de novo design of new Bcr-Abl allosteric inhibitors.

## Figures and Tables

**Table 1:** Representative examples of 15 top-ranked potential inhibitors generated through automated de novo drug design. The superscript letters a, b and c indicate the docking scores (kcal/mol), multi-objective S(x) fitness values, and Synthetic Accessibility (SA) score, respectively.

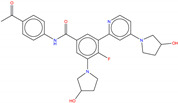	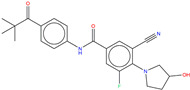	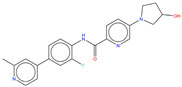
Bcr-Abl.lib_01-10.704 ^a^ 3.791 ^b^ 3.977 ^c^	Bcr-Abl.lib _02-10.338 ^a^ 3.743 ^b^ 2.970 ^c^	Bcr-Abl.lib_03-10.305 ^a^ 3.737 ^b^ 2.810 ^c^
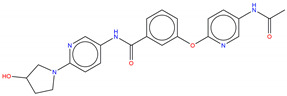	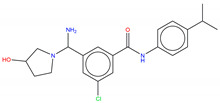	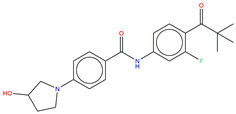
Bcr-Abl.lib_04-10.296 ^a^ 3.874 b 2.764 ^c^	Bcr-Abl.lib_05-10.246 ^a^ 3.667 ^b^ 4.055 ^c^	Bcr-Abl.lib_06-10.169 ^a^ 3.795 ^b^ 2.643 ^c^
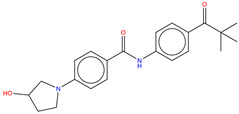	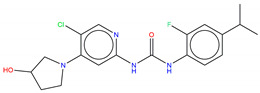	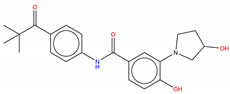
Bcr-Abl.lib_07-10.158 ^a^ 3.914 ^b^ 2.475 ^c^	Bcr-Abl.lib_08-10.139 ^a^ 2.998 ^b^ 2.898 ^c^	Bcr-Abl.lib_09-10.104 ^a^ 3.909 ^b^ 2.689 ^c^
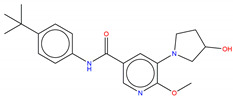	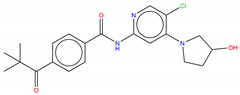	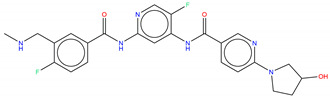
Bcr-Abl.lib_10-10.072 ^a^ 3.989 ^b^ 2.749 ^c^	Bcr-Abl.lib_11-10.052 ^a^ 3.727 ^b^ 2.869 ^c^	Bcr-Abl.lib_12-9.991 ^a^ 2.986 ^b^ 3.472 ^c^
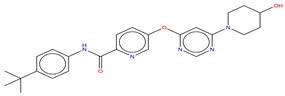	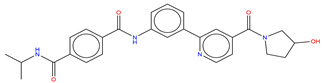	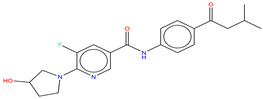
Bcr-Abl.lib_13-9.939 ^a^ 3.506 ^b^ 2.501 ^c^	Bcr-Abl.lib_14-9.916 ^a^ 3.437 ^b^ 2.668 ^c^	Bcr-Abl.lib_15-9.915 ^a^ 3.979 ^b^ 2.735 ^c^

## Supplementary Materials

The supplementary materials are available online. Note that SA values are reported as mean along with standard deviation. File_SI.csv.
