# Supplementary material for: Bcr-Abl Allosteric Inhibitors: Where We Are and Where We Are Going to
Source: Molecules. 2020 Sep 14;25(18):4210. doi: 10.3390/molecules25184210 (PMC7570842; doi:10.3390/molecules25184210)
Supplement: Supplementary file 1 [file molecules-25-04210-s001.zip › molecules-909766-supplementary materials-english done.docx]

SUPPORTING INFORMATION

Bcr-Abl Allosteric Inhibitors: Where we are and where we are going to

Francesca Carofiglio ^1^^,†^ Daniela Trisciuzzi ^1,2,†^ Nicola Gambacorta ^1^ Francesco Leonetti ^1^ Angela Stefanachi ^1,^* and Orazio Nicolotti. ^1,^*

^1^ Dipartimento di Farmacia Scienze del Farmaco;

^2^ Molecular Horizon srl, Via Montelino 32, 06084 Bettona, Italy;

[francescacarofiglio94@gmail.com](mailto:francescacarofiglio94@gmail.com)(F.C.); [daniela.trisciuzzi@uniba.it](mailto:daniela.trisciuzzi@uniba.it%20) (D.T.); [nicola.gambacorta1@uniba.it](mailto:nicola.gambacorta1@uniba.it)(N.G.); [francesco.leonetti@uniba.it](mailto:francesco.leonetti@uniba.it)(F.L.); [angela.stefanachi@uniba.it](mailto:angela.stefanachi@uniba.it)(A.S.); (O.N.) orazio.nicolotti@uniba.it.

***** Correspondence: [angela.stefanachi@uniba.it](mailto:angela.stefanachi@uniba.it) Tel.: +390805442783 (A.S.); orazio.nicolotti@uniba.it. Tel.: +390805442551 (O.N.)

**^†^** These two authors contributed equally

**Table S1.** List of residues along with their energetic contributions of myristate binding site for three Abl crystal structures co-crystallized with myristic acid (PDB entry, 1OPK), GNF-2 (PDB entry, 3K5V), and Abl001 (PDB entry, 5MO4), respectively. CRY-, N1-, and O-GRID probes are computed by using BioGPS software (version, company, city, the abbreviation of state if USA, country).

|  | **Myristic acid** | | | **GNF-2** | | | Abl001 | | |
| --- | --- | --- | --- | --- | --- | --- | --- | --- | --- |
| **Res** | **CRY** | **N1** | **O** | **CRY** | **N1** | O | CRY | N1 | **O** |
| Arg351 | –0.394 |  |  | –0.619 |  | –1.094 | –1.349 | –1.593 | –4.683 |
| Ser355 |  |  |  |  | –2.170 |  |  |  |  |
| Ala356 | –1.712 | –7.263 | –1.492 | –1.775 | –7.360 | –1.472 | –1.318 | –7.343 | –1.537 |
| Leu359 | –1.496 | –4.930 | –1.847 | –2.643 | –4.525 | –1.543 | –1.511 | –4.342 | –1.867 |
| Leu360 | –4.366 | –3.347 | –1.762 | –3.595 | –4.114 | –1.692 | –4.120 | –3.551 | –1.826 |
| Ala363 | –2.509 |  | –2.003 | –3.349 |  | –1.836 | –1.483 | –1.714 | –2.006 |
| Leu448 | –2.610 | –5.35 | –1.843 | –1.325 | –7.535 | –1.378 | –1.625 | –7.599 | –1.723 |
| Ile451 | –1.614 | –2.761 | –1.607 | –2.549 | –2.605 | –1.308 | –2.938 | –2.414 | –1.647 |
| Ala452 | –2.711 | –7.518 | –1.411 | –2.328 | –6.444 | –1.519 | –3.390 | –7.426 | –1.959 |
| Thr453 |  |  |  |  |  |  | –1.495 | –6.004 | –1.569 |
| Tyr454 | –1.13 | –4.067 | –2.901 | –1.292 | –1.971 | –6.453 | –1.399 | –1.284 | –2.736 |
| Met456 |  |  |  |  |  |  | –1.301 | –1.277 | –1.417 |
| Pro480 |  |  |  |  |  |  | –1.521 |  |  |
| Glu481 | –1.252 | –7.127 | –1.102 | –2.736 | –7.115 |  | –1.653 | –7.934 | –4.244 |
| Gly482 | –1.396 | –4.648 | –1.426 | –1.417 | –2.851 |  | –1.337 | –1.344 |  |
| Cys483 | –2.743 | –3.892 | –2.395 | –1.551 | –3.708 | –3.100 | –2.638 | –3.684 | –3.323 |
| Pro484 | –1.416 |  | –1.421 | –1.315 |  |  | –1.343 |  | –1.332 |
| Val487 | –2.363 | –2.646 | –1.452 | –2.742 | –2.597 | –1.316 | –2.722 | –2.932 | –1.656 |
| Phe512 | –1.291 |  | –1.035 | –1.523 |  |  | –2.453 | –2.153 | –1.705 |
| Ile521 | –2.584 | –2.404 | –1.757 | –1.621 | –2.093 | –1.301 | –2.667 | –2.428 | –1.664 |
| Val525 | –1.738 |  | –1.459 | –2.173 |  | –1.224 | –1.505 | –1.888 | –1.079 |
| Leu529 | –1.911 | –1.189 | –1.123 | –1.531 |  |  | –1.034 |  |  |
| HOH |  |  |  | –1.308 | –6.777 | –6.593 | –1.402 | –7.114 | –6.181 |
| HOH |  |  |  | –0.921 | –6.255 | –8.942 | –1.066 | –6.226 | –7.466 |

**Table S2.** Features selected for pair based multi-objective optimization generation of the targeted Bcr-Abl chemical library.

| **Features** | **Min** | **Max** | **Pareto** |
| --- | --- | --- | --- |
| MW | 337 | 562 | Minimize |
| logP | 2.6 | 4.3 | Minimize |
| similarity | 0.3 | 0.9 | Maximize |

**Table S3.** Quality metrics of the targeted chemical library generated by de novo drug design. Note that SA values are reported as mean along with standard deviation.

| **Targeted chemical library** | **Validity** | **Unicity** | **IntDiv** | **Filters** | **SA** |
| --- | --- | --- | --- | --- | --- |
| BCR-ABL library | 0.997 | 0.994 | 0.605 | 0.828 | 2.704±0.232 |

As reported in Table S2, the Bcr-Abl library reveals good values of the quality metrics. Moreover, 99.7% of the generated molecules are valid, and 99.4% are unique, showing an internal diversity with a percentage equal to 60.5%. An average synthetic accessibility value equal to 2.704 ± 0.232 expressing a low molecular complexity of de novo library.

**Figure S1.** Average value of the S(x) fitness function computed at each iteration of the pair based multi-objective optimization algorithm for the Bcr-Abl case study.


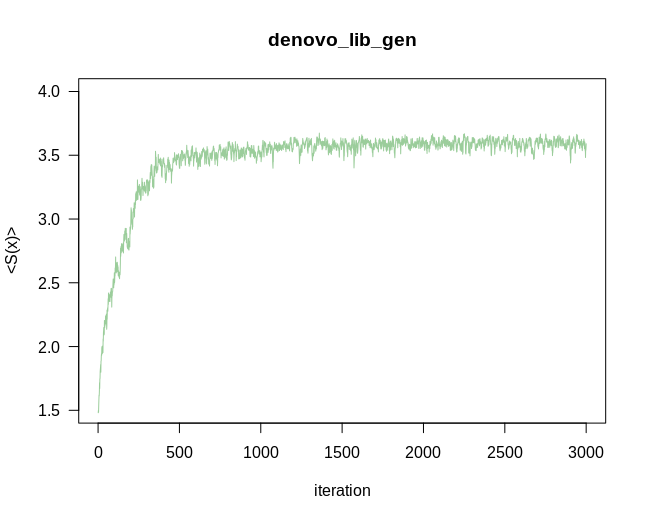


The pair based multi-objective optimization algorithm progressed through 3000 cycles, by generating 500 compounds per iteration. The targeted chemical library was built sampling 1000 potential Bcr-Abl inhibitors from the policy that maximizes the average of S(x) fitness values (that is <S(x)>) and was assessed by calculating the following quality metrics: (a) Validity, which represents the fraction of chemically valid SMILES; (b) unicity, which stands for a fraction of unique generated SMILES; (c) Internal Diversity (IntDiv), which accounts for the overall molecular diversity of the targeted chemical library; (d) filters, which reflect the fraction of de novo generated molecules devoid of pan-assay interference compounds (PAINS) alerts and of medicinal chemistry filters (MCFs); ( e) Synthetic Accessibility (SA) score, which provides a heuristic estimate of how hard (SA = 10) or how easy (SA = 1) is the chemical synthesis of a given molecule (see table S3). A synoptic assessment of the de novo designed targeted chemical library is provided in Table S2 by reporting the mentioned quality metrics.


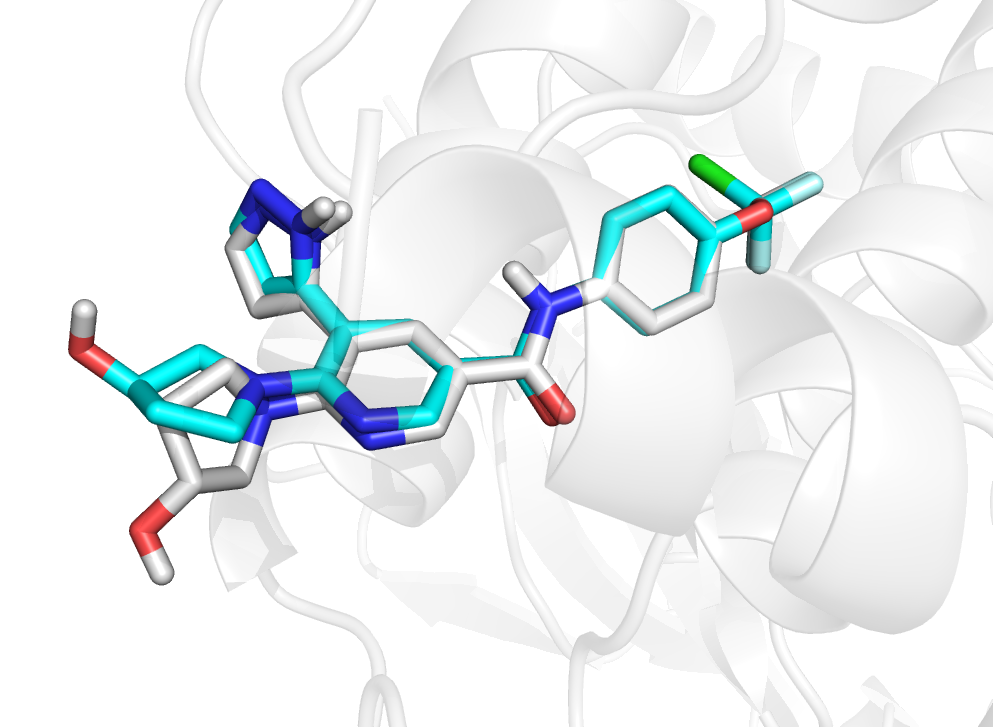


**Figure S2.** Overlap of X-ray solved (gray sticks) and top-scored docking (cyan sticks) poses for ABL001 (RMSD = 1.020 Å).
